# A Retrospective Review of Anthralin in Petrolatum in the Treatment of Alopecia Areata in the Pediatric Population

**DOI:** 10.1177/12034754231191060

**Published:** 2023-08-09

**Authors:** Yaron Zafrir, Kimberly Tantuco, Ang J. A. Tangtatco, Nhung Ho

**Affiliations:** 1 7979 Sickkids Hospital, Department of Pediatrics, Division Dermatology, University of Toronto, Canada

**Keywords:** alopecia areata, anthralin, pediatric patients

## Abstract

**Background/Objectives:**

Alopecia areata (AA) is a T-cell driven autoimmune disease, which results in hair loss. This study aims to determine the efficacy, tolerability and safety of different concentrations of anthralin in the treatment of pediatric AA.

**Methods:**

A retrospective cohort study of patients < 18 yo diagnosed with AA treated with anthralin at SickKids Hospital, Toronto dermatology outpatient clinic in 2016 - 2018. Anthralin used at 0.1%, 0.2%, 0.5% and 1% in petrolatum at short contact, at increments of 15 minutes every week until a 1 hr maximum contact achieved. No other treatment was used in conjunction. Severity of Alopecia Tool (SALT) scores (SS) were determined using photographs and descriptions to assess severity of alopecia at baseline and post anthralin treatment.

**Results:**

A total of 11 charts were reviewed in this retrospective cohort. Hair loss pattern; 3 patients with patchy, 6 had mixed (patchy and ophiasis), and 2 were totalis. All except for 1 patient had failed traditional treatments. One patient had complete hair regrowth, 3 showed more than 85% hair re-growth and 7 patients showed more than 75% hair regrowth, the average time for this to occur was 6.5 months. None of the patients experience serious side effects.

**Conclusions:**

Our study demonstrated the efficacy and tolerability of topical anthralin 0.1% to 1% in pediatric alopecia areata. In our study, anthralin 0.2% appears to offer the best performance and tolerability profile among the different concentrations used, with treatment course of at least 6 months in order to achieve more than 75% hair regrowth.

## Introduction

Alopecia areata (AA) is a T-cell-mediated autoimmune disease that targets the hair follicle resulting in patchy, nonscarring hair loss. It has been associated with autoimmune diseases, presence of atopy, genetic predisposition and environmental trigger^1,2^ This disease affects both adults and children but AA in the pediatric population has been associated with poorer prognosis.^3^ Literature reports that 34% to 50% of patients with AA resolve spontaneously within a year. However, 15% to 25% will progress to total hair loss of the scalp or total hair loss of the scalp and body, with unlikely complete resolution.^4,5^ Alopecia areata has been associated with significant psychosocial sequel and a poorer quality of life in children. Therefore, treatment is often pursued.^6^ Many treatment modalities are available, however, there is no universally accepted treatment that produces complete cure and sustains remission.^7^

Anthralin is a derivative of chrysaborin, prepared from the araroba tree in Brazil over a century ago.^8^ It has been shown to be efficacious in achieving hair regrowth in patients with alopecia areata.^9-11^ Proposed mechanisms for efficacy in AA are its antiinflammatory properties^12^ and ability to cause an irritant contact dermatitis that results in hair regrowth.^13^ Anthralin 1% ointment has been reported to be effective and tolerable for AA in children. This study aims to evaluate the efficacy, determine tolerability and safety of different concentrations of anthralin in the treatment of pediatric AA.

## Materials and Methods

We performed a retrospective review of pediatric patients with alopecia areata treated with anthralin at the Hospital for Sick Children, Toronto from 2016 - 2018. An informed consent was obtained. The inclusion criteria were defined: age ≤18 years, diagnosed clinically with alopecia areata, and initiated treatment with anthralin at baseline visit. Baseline visit was considered the day anthralin was initiated. Exclusion criteria were defined: anthralin was initiated prior to baseline assessment, unable to follow up and concomitant use of other treatment for AA. Anthralin was used at 0.1%, 0.2% and 0.5% in petrolatum and 1% at short contact, at increments of 15 minutes every week until a 1 hr maximum contact achieved. The next higher concentration was proposed to patient at subsequent visit if no significant response and acceptable tolerance were obtained. No other treatment was used in conjunction. Demographic, clinical characteristics and Severity of Alopecia Tool (SALT) scores (SS) of the patients were recorded at baseline, each follow up and post anthralin treatment. Treatment effect was measured by percent change in SS from baseline. Disease percentage (SALT score) was calculated as the sum of the scores of the percentage of hair loss in each of the four areas of the scalp multiplied by the weighing factor representing the percentage of that area (0.18 or 18% for each R and L side, 0.40 or 40% for the vertex and 0.24 or 24% for the posterior area). The SALT score is 0 if the scalp has no hair loss and 100 if total hair loss.

In evaluation of percentage recovery in alopecic plaques, existing percentage hair loss was used as the baseline value. For example, for a patient referred with a percentage hair loss of 75% at the beginning of treatment and for whom the size of the alopecic patch was 50% at the end of treatment, then percentage recovery = (75–50) ⁄ 75 = 0.33 = 33%. When calculating percentage recovery, the presence of pigmented terminal hair only was used to determine the baseline value.

## Results

A total of 11 charts were reviewed in this retrospective cohort, summarized in Table 1. The sex ratio was seven females to four males. All the patients had brown or black hair. Mean age of AA onset was 5.9 years old, of current AA episode was 6.6 years old, and of initiation with anthralin was 9.9 years old. The pattern of hair loss was identified 3 were patchy, 6 had mixed (patchy and ophiasis), and 2 were totalis. All except for 1 patient had failed traditional treatments.

**Table 1:** Summarizes the Demographic and Clinical Characteristics of Our Cohort.

PT	Gender	Hair color	Age, Y			Previous treatments	Pattern	Medical history	SALT % at base line	SALT % at best result	Change
			Onset	Current patient at center	Initiation of anthralin						
1	f	Black	6	9	13	CS, TCI, ILSI, DPCP, MTX	Mix	Mild autism	40	6.5	84
2	f	Black	6	8	8	None	Mix	Eczema	50	0	100
3	f	Brown	6	6	9	CS	Mix		70	7	90
4	f	Black	13	13	17	CS, TCI, ILSI	Mix		100	13	87
5	m	Black	13	14	17	CS, TCI	Totalis	Iron deficiency anemia	100	3	94
6	m	Black	1	1	11	CS, TCI, ILSI, DPCP, combination	Patchy		14	4	71
7	f	Brown	4	4	6	CS	Patchy		62	7	89
8	f	Brown	6	6	10	CS	Mix	Hypothyroid	19	4	79
9	m	Black	2	4	6	CS, TCI	mix		66	26	61
10	m	Black	2	2	4	CS	patchy		85	68	20
11	f	Black	6	6	8	CS, TCI, OCS, MTX Combination	Totalis		100	100	0

Abbreviations: CS, Corticosteroids; DPCP, diphenylcyclopropenone; F, female; ILSI, intralesional corticosteroid injections; M, male; MTX, methotrexate; oral corticosteroids, OCS; topical calcineurin inhibitors, TCI.

Alopecia pattern were classified as follows - Patchy, totalis, mixed- patchy +ophiatic

**Figure 1:**
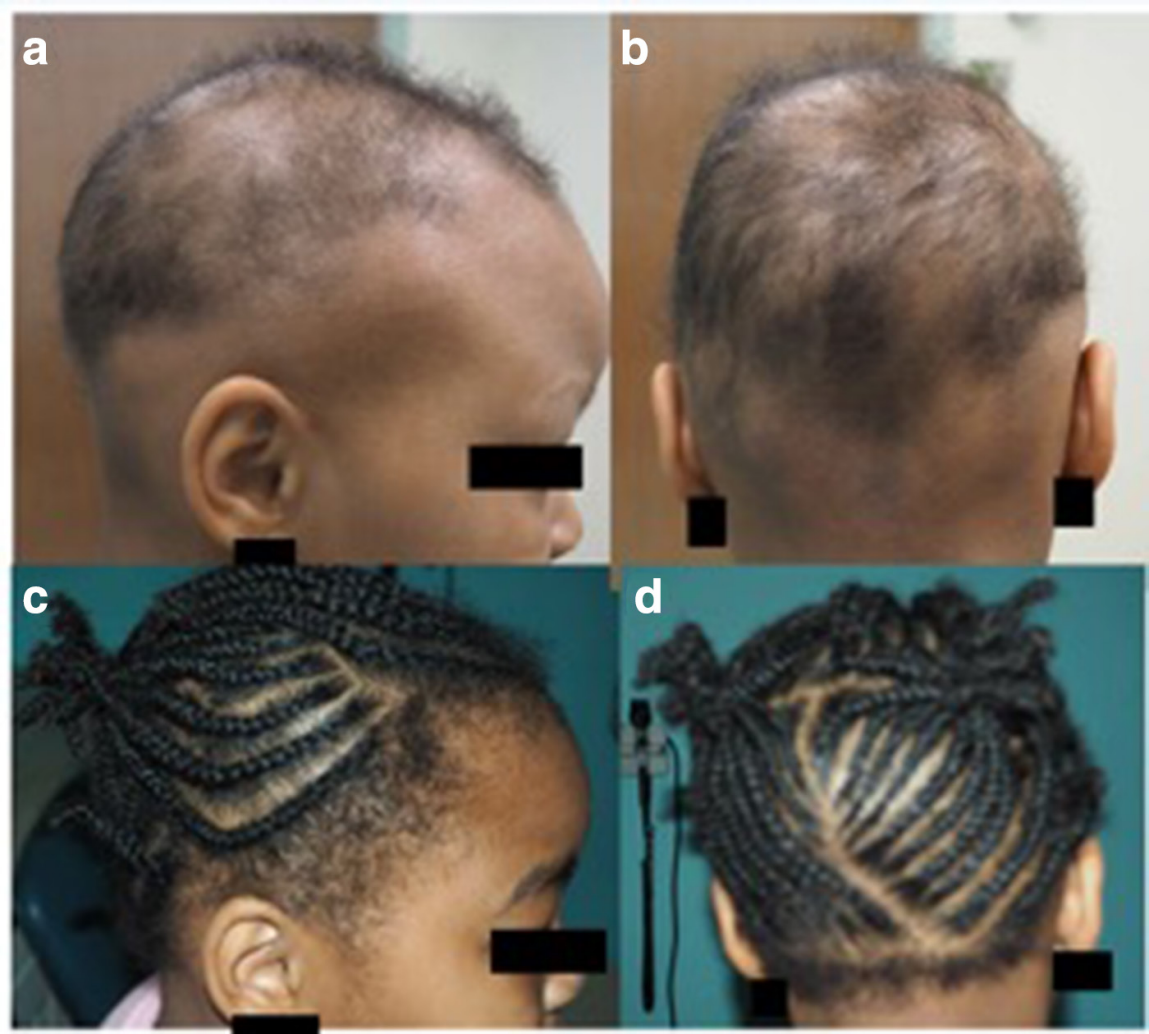
Response to anthralin. Patient 2 at baseline (**a, **b) and after (**c, **d).

**Figure 2:**
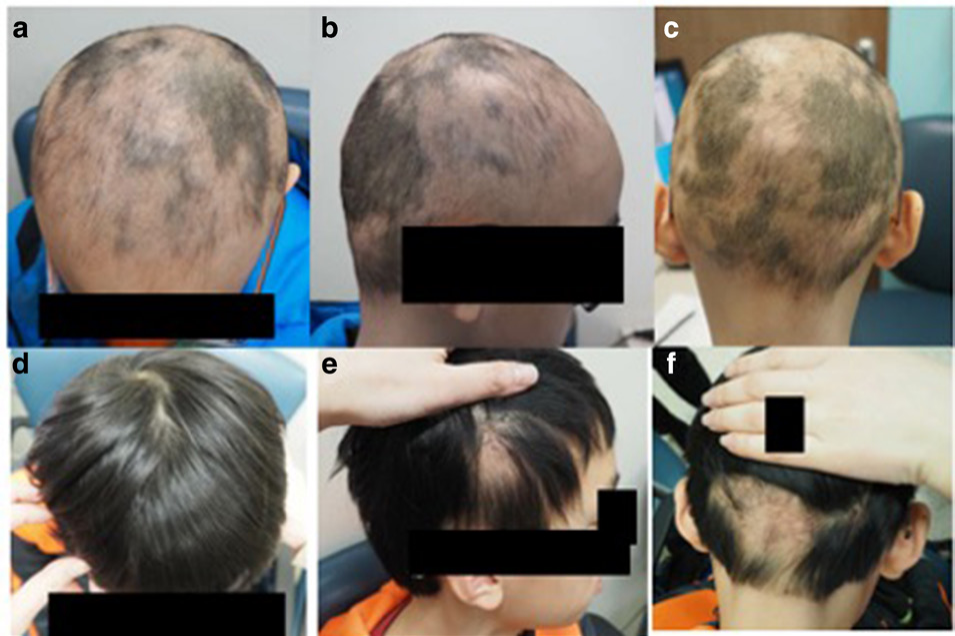
Response to anthralin. Patient 5 baseline (**a, b, **c) and after (**d, e, **f).

Mean SS at baseline was 64%, mean SS at best results was 28.4%, and the mean percent change in SS was 58.8%. One out of the 11 patients had 100% complete hair growth and 3 showed more than 85% hair re-growth (Figures 1 and 2). 7 patients showed more than 75% hair regrowth, the average time for this to occur was 6.5 months. Table 2 shows the length of time anthralin treatment was necessary to achieve a response and the time to get to more than 75%. 81% percent (9/11) had more than 50% change in SS. Eighty-one percent of the patient had a response to anthralin at 0.1%.

**Table 2:** Anthralin Concentrations That Were Applied and Time to >75% hair Regrowth.

Patient	Concentration	Time to first response in months	Concentration	Time in months	Concentration	Time in months	Time to get to >75% in months
1	0.1	2	0.2	10	0.5	7	4
2	0.1	5					3
3	0.1	2	0.2	7			9
4	0.1	6	0.2	5	0.5	10	10
5	0.1	2	0.2	6	0.5	3	6
6	0.1	2	0.5	9			
7	0.1	2	0.5	8	1	4	5
8	0.1	3	0.2	6	0.5	2	9
9	0.1	6	0.2	6	0.5	3	
10	0.1	1	0.2	7			
11	0.1	3	0.2	1	0.5	4	

Patient 2 had patchy alopecia and anthralin 0.1% was given as first line therapy at the onset of her alopecia. She had 100% hair regrowth by 9 months while on anthralin 0.1% (Figure 1). This was sustained even after 6-mo follow up. Patient 11 with alopecia totalis did not respond to treatment with anthralin 0.1%, 0.2% and 0.5%. None of the patients experience serious side effects. All patients had a brown discoloration of the scalp from Anthralin. Some had mild scaliness. Irritation^3^ and pruritus^1^ were the only side effected noted. One patient developed irritation on anthralin 0.5% and was then decreased to 0.2%. At 0.2%, no adverse effects reported. Another patient developed post auricular dermatitis while on 0.5% anthralin and this resolved with topical corticosteroids. None needed to discontinue treatment.

## Discussion

Anthralin for the treatment of alopecia areata was first described in 1979. The irritant contact dermatitis was a desired reaction that brought about hair regrowth.^13^ Tang also suggested that free radicals produced by anthralin mount an antiinflammatory effect in alopecia areata. This study also showed decreased expression of the proinflammatory cytokine TNF.^12^

Anthralin has been shown to be effective in achieving hair regrowth in patients with alopecia areata^9-11^ There have been no reports of serious side effects. The most commonly reported adverse effects are pruritus and mild local irritation.^10,11^ It is well tolerated and has a limited risk of serious side effects making it a good treatment option for pediatric population. However, there is a paucity of data^11,14,15^ and total of 69 patients, that demonstrated complete response rates that are ranging between 32% and 33.3% and relapse rates of 9.5% to 64% with mean time to maximal response ranged between 9 to 15 months.^16^

A study that was conducted by Wu et. al^14^ reviewed 37 pediatric patients with AA who were started on topical anthralin, some naïve and some in conjunction with traditional therapy showed 68% (25/37) to have at least 50% maximal scalp regrowth. Thirty-two percent (12/37) had complete scalp hair regrowth. The mean time to first response was 3.4 months. Four patients discontinued therapy due to irritation.^14^ In our study, using anthralin alone, 73% percent (8/11) had more than 50% hair regrowth. The mean time to first response was 3 months as similarly reported by Wu et. Al.^14^

In a study that was performed by Ozdemir et al.^11^ with anthralin 1% ointment on 1 side of the scalp showed 50% reduction in their pretreatment alopecia severity score at 9 months of treatment in children with chronic, severe, treatment refractory and extensive alopecia areata.^11^ Our study showed that anthralin 0.2% was the highest concentration used at the time the patients reached 75% hair regrowth. Early intervention yielded the best result (patient 2), as the delay in intervention would prohibit response (patient 6).

The use of anthralin in combination with Leflunomide was reported to be beneficial in a case report of a recalcitrant ophiasis-pattern AA who had failed steroid therapy.^15^ The authors reported a complete response within 3 months, they suggested that this outcome might be attributed to the action of leflunomide and anthralin on the JAK-STAT pathway.^15^

A recent systemic review which evaluated various method such as immunotherapy, light/laser therapy, and contact dermatitis induced by anthralin, concluded that although anthralin showed the lowest rate of hair regrowth, anthralin -related acceptable responses were the most durable and sustained ones^17^

Side effects to anthralin include lymphadenopathy, hyperpigmentation and local irritation, with all resolving following cessation of treatment.^18^ Local irritation is the main limitation of anthralin usage and in some cases result in discontinuing the medication.^14^ All of our 11 patients completed their recommended treatment protocol and non were required to discontinue treatment. The use of anthralin in combination with other topical treatments should be carefully considered as it may result in an increased irritation with no demonstration of increase in effectiveness.^19^

## Conclusion

Our study was able to demonstrate the efficacy and tolerability of topical anthralin 0.1% to 1% in pediatric alopecia areata. The limitation of anthralin is local irritation. In our study, anthralin 0.2% appears to offer the best performance and tolerability profile among the different concentrations used. Treatment course may need to be continued for at least 6 months in order to achieve significant (more than 75%) hair regrowth. Limitation of this study is a small sample size and no standardization. Prospective study with larger sample size and longer follow up period, that will study the effects of this treatment on quality of life in these alopecia areata patients should be considered.
